# LIP5, a MVB biogenesis regulator, is required for rice growth

**DOI:** 10.3389/fpls.2023.1103028

**Published:** 2023-01-17

**Authors:** Mengxue Wang, Shuwei Luo, Baofang Fan, Cheng Zhu, Zhixiang Chen

**Affiliations:** ^1^ College of Life Science and Key Laboratory of Marine Food Quality and Hazard Controlling Technology of Zhejiang Province, China Jiliang University, Hangzhou, China; ^2^ Department of Botany and Plant Pathology and Center for Plant Biology, Purdue University, West Lafayette, IN, United States

**Keywords:** multivesicular bodies, LYST-INTERACTING PROTEIN5, SKD1, rice, endocytosis, cellular homeostasis, plant growth

## Abstract

LYST-INTERACTING PROTEIN5 (LIP5) is a conserved regulator of multivesicular body (MVB) biogenesis in eukaryotes. In Arabidopsis, AtLIP5 is a target of stress-responsive MITOGEN-ACTIVATED PROTEIN KINASE3 and 6 and mediates stress-induced MVB biogenesis to promote stress responses. However, Arabidopsis *atlip5* knockout mutants are normal in growth and development. Here we report that rice *OsLIP5* gene could fully restore both the disease resistance and salt tolerance of the Arabidopsis *oslip5* mutant plants to the wild-type levels. Unlike Arabidopsis *atlip5* mutants, rice *oslip5* mutants were severely stunted, developed necrotic lesions and all died before flowering. Unlike in Arabidopsis, LIP5 regulated endocytosis under both stress and normal conditions in rice. These findings indicate that there is strong evolutionary divergence among different plants in the role of the conserved LIP5-regulated MVB pathway in normal plant growth.

## Introduction

Multivesicular bodies (MVBs) are late endosomes in the endocytic pathway (Cui et al., 2016). MVBs also mediate protein trafficking to the vacuole in the secretory pathway and are also called prevacuolar compartments in plants (Cui et al., 2016). MVBs are formed from trans-Golgi network/early endosomes by the invagination and budding of the limiting membrane into the lumen through the action of protein complexes named ESCRTs (endosomal sorting complexes required for transport) (Piper and Katzmann, 2007). MVB biogenesis is essential for plant growth and development and mutants for essential ESCRT components and associated factors such as SKD1 (SUPPRESSOR OF K^+^ TRANSPORT GROWTH DEFECT1) ATPase, which catalyzes the disassembly of the ESCRT III complex during MVB biogenesis, are lethal (Haas et al., 2007; Zhang et al., 2013; Gao et al., 2014). LYST-INTERACTING PROTEIN 5 (LIP5) is a conserved regulator of MVB biogenesis by activating the SKD1 ATPase (Haas et al., 2007; Skalicky et al., 2012; Guo and Xu, 2015; Vild et al., 2015). Importantly, Arabidopsis knockout mutants for *AtLIP5* have a largely normal phenotype in growth and development, indicating that the basal SKD1 activity is sufficient for MVB biogenesis under normal growth conditions (Haas et al., 2007; Wang et al., 2014; Wang et al., 2015). However, the *atlip5* mutants are compromised both in stress-induced MVB formation and in disease resistance and stress tolerance (Wang et al., 2014; Wang et al., 2015; Xia et al., 2016). Arabidopsis AtLIP5 is subjected to degradation by the proteasome system under normal growth conditions but becomes stable under stress conditions upon phosphorylation by stress-responsive MITOGEN-ACTIVATED PROTEIN KINASE3 and 6 (MAPK3 and 6) to promote stress-induced MVB biogenesis (Wang et al., 2014; Wang et al., 2015). Thus, Arabidopsis AtLIP5 is a key regulator of stress-induced MVB biogenesis. Here, we have identified the functional homologue of the *LIP5* protein in rice through complementation of the Arabidopsis *atlip5* mutant plants. Furthermore, we have generated loss-of-function mutants for rice *OsLIP5* gene through CRISPR/Cas9. Surprisingly, the rice *oslip5* mutants were severely stunted, developed necrotic lesions and all died before flowering. Unlike in Arabidopsis, OsLIP5 regulated endocytosis under both stress and normal conditions in rice roots. The drastic difference in the *lip5* mutant phenotypes between Arabidopsis and rice raises an important question about the divergent functionality of LIP5-activated MVB pathway in plant growth and development under normal growth conditions.

## Materials and methods

### Plant materials and growth conditions

Arabidopsis (*Arabidopsis thaliana*) mutant and WT plants used in the study are all in the *Col-0* background. The *atlip5* mutants have been previously described (Lai et al., 2011; Wang et al., 2014). Arabidopsis were grown in growth chambers or rooms at 24°C, 120 µmol m^-2^s^-1^ light on a photoperiod of 12-hour light and 12-hour dark. Rice mutant and WT plants are in the background of rice cultivar Zhonghua 11. Rice plants were grown hydroponically in a modified rice culture solution containing 1.425 mM NH_4_NO_3_, 0.2 mM NaH_2_PO_4_, 0.513 mM K_2_SO_4_, 0.998 mM CaCl_2_, 1.643 mM MgSO_4_, 0.009 mM MnCl_2_, 0.075 mM (NH_4_)_6_Mo_7_O_24_, 0.019 mM H_3_BO_3_, 0.155 mM CuSO_4_, and 0.152 mM ZnSO_4_ with 0.125 mM EDTA-Fe. pH 5.5 (Yoshida et al., 1976). Rice plants were grown in a growth room with a 14 h day (28°C)/10 h night (23°C) photoperiod at approximately 200 μmol m^-2^ s^-1^ photon density, and approximately 60% humidity as described previously (Xu et al., 2017).

### Identification rice *OsLIP5* gene and analysis of OsLIP5 subcellular localization

Rice OsLIP5 gene was identified from the rice genome by BLASP search using Arabidopsis OsLIP5 as a query with an e-value cutoff at 1e-10. For subcellular localization of OsLIP5, full-length *OsLIP5* coding sequence was PCR-amplified from the rice cDNA using *OsLIP5*-specific primers (AGCCCATGGGGAGCGACGCGGAG and AGCTTAATTAAATGAGTTTCGGCGGAAGG) and fused to the *GFP* gene behind the *CaMV 35S* promoter in a pFGC5941-derived binary plant expression vector (Li et al., 2021). Constructs for ARA6-mRFP and dexamethasone-inducible *SKD1* have been described previously (Wang et al., 2014). Colocalization analysis of AtLIP5 and OsLIP5 with MVB maker ARA6 in *Nicotiana benthamiana* was performed as previously described (Wang et al., 2014).

### Analysis of LIP5-SKD1 interactions using yeast two-hybrid assays

Full-length OsLIP5 coding sequence was PCR amplified using gene-specific primers and cloned into yeast two-hybrid bait pAD-GAL4 vector as previously described (Wang et al., 2014). pBD-AtSKD1 bait and pAD-AtLIP5 prey constructs have been previously described (Wang et al., 2014). Various combinations of bait and prey constructs were co-transformed into yeast cells and interaction were analyzed by assaying *LacZ* β-galactosidase activity as described previously (Wang et al., 2014).

### Complementation in Arabidopsis

For functional analysis through complementation of Arabidopsis *atlip5* mutant plants, the coding sequence of *OsLIP5* was PCR-amplified from rice cDNA using *OsLIP5*-specific primers and cloned into a pFGC5941-derived binary plant expression vector between the *CaMV 35S* promoter and a 4xmyc epitope tag (Li et al., 2021). The *OsLIP5* expression construct was introduced into the Arabidopsis *atlip5-1* mutant plants using the floral dipping method (Clough and Bent, 1998). Transgenic Arabidopsis plants were identified by their herbicide resistance to Basta. Expression of the transgene in the transgenic plants was analyzed by protein blotting using anti-myc monoclonal antibodies as previously described (Li et al., 2021).

### Protein blotting

Protein isolation, electrophoretic separation, blotting and detection of LIP5-myc proteins using anti-myc antibodies were performed as previously described (Li et al., 2021).

### Disease resistance and salt tolerance assays of Arabidopsis plants

Assays of Arabidopsis plant resistance to a virulent strain of *Pseudomonas syringae* pv *tomato* DC3000 (*Pst*DC3000) and tolerance to salt stress (150 mM NaCl) were performed as previously described (Wang et al., 2014; Wang et al., 2015).

### Generation of Rice *OsLIP5* gene mutations suing CRISPR/Cas9 genome editing

Two sites on the first and third exons of OsLIP5, both of which are close to the 5’-end of the *OsLIP5* coding sequence, were selected as targets for genome editing. The target sequences (ggcaTCGGCTCTACGCGATGGAGA/aaacTCTCCATCGCGTAGAGCCGA and ggcgGCAGACAAACAGGATCGTGC/aaagCACGATCCTGTTTGTCTGC) were inserted a rice CRISPR/Cas9 vector as previously described (Liu et al., 2020). Rice transformation into rice variety Zhonghua 11 was performed by co-cultivation of *hst1* rice calli with *Agrobacterium tumefaciens* strain EHA105 containing the CRISP/cas9 construct as previously described (Hiei et al., 1994). For identification of mutations in the target sites, the regions were PCR-amplified using PCR primers flanking the regions from independent T1 transgenic lines and directly sequenced.

### Analysis of endocytic activity in rice roots

Fluorescence microscopy of FM1-43 (Life Technologies) internalization in the roots of 7 days-old rice seedlings was also performed as described previously (Wang et al., 2014), with minor changes of the concentration of FM1-43 and staining time reduced to 10 μM and 20 min, respectively. The wavelength settings of confocal microscopy were as follows: excitation at 488 nm and emission at 600 to 650 nm for FM1-43,

## Results and discussion

### OsLIP5 protein sequence, subcellular localization and interaction with SKD1

**Figure 1:**
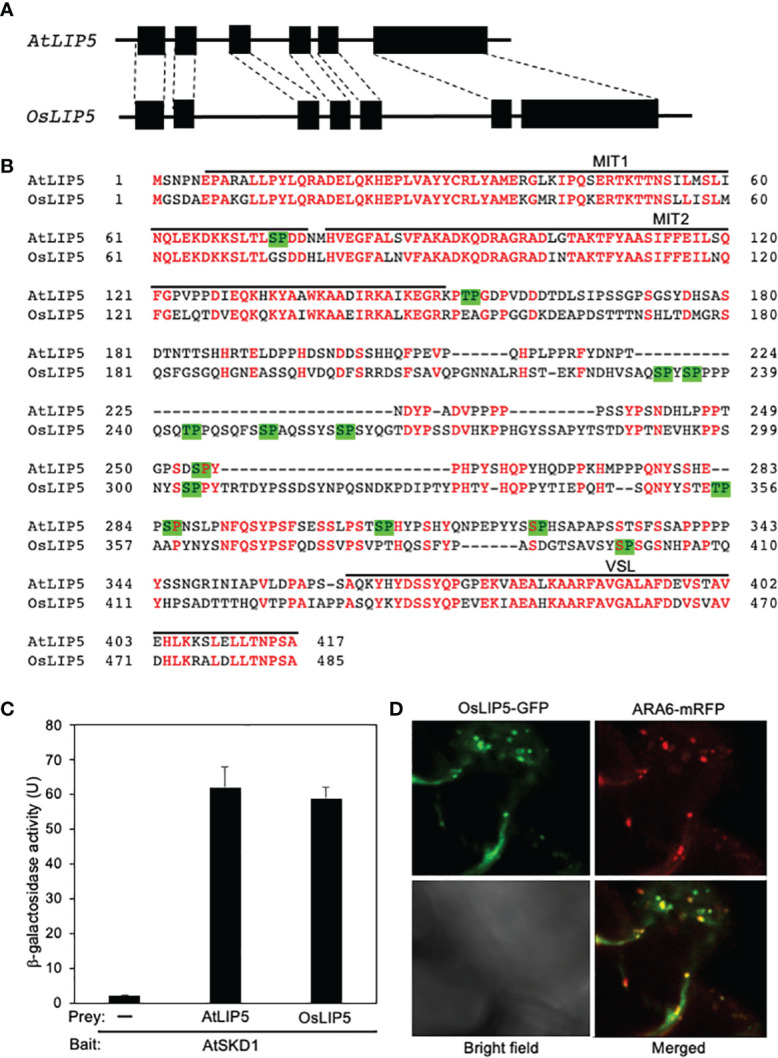
Gene structure, protein sequence, interaction with SKD1 and subcellular localization of rice LIP5. **(A)** Arabidopsis and rice *LIP5* gene intron (line) and exon (box) architecture. The homologous exon between the Arabidopsis and rice LIP5 genes are indicated by dashed lines. **(B)** Amino acid sequence alignment of Arabidopsis and rice LIP5 proteins. Identical amino acid residues are in red. The N-terminal tandem MIT and C-terminal VSL domains are indicated. Putative TP and SP phosphorylation sites by proline-directed protein kinases are in green background. **(C)** Yeast two-hybrid assays of LIP5 proteins with AtSKD1. The indicated fusion bait and prey constructs were co-transformed into yeast cells. Empty pAD-Gal4 vector was used as negative control (-). Yeast transformants were analyzed for LacZ reporter gene expression through assays of β-galactosidase activity using ONPG (o-nitrophenyl-β-D-galactopyranose) as substrate. Data of arbitrary units (U) represent means and standard errors (n=5). **(D)** Subcellular colocalization of OsLIP5-GFP with ARA6-mRFP when co-expressed with AtSKD1 in the leaf epidermal cells of *N. benthamiana*.

As in Arabidopsis, there is a single *LIP5* gene in the rice genome. The intron-exon architecture of rice *OsLIP5* is very similar to that of Arabidopsis *AtLIP5* (**Figure 1A**). The rice OsLIP5 protein is also structurally highly homologous to Arabidopsis AtLIP5 with 54% identify and 62% similarity in protein sequence (**Figure 1B**). LIP5 binds a subset of ESCRT-III proteins through its N-terminal tandem microtubule-interacting and trafficking (MIT) domain and binds SKD1 ATPase through its C-terminal VSL (Vta1/SBP1/LIP5) domain (Haas et al., 2007; Spitzer et al., 2009; Skalicky et al., 2012; Wang et al., 2014). Sequence similarity is particularly high between AtLIP5 and OsLIP5 at these N- and C-terminal domains (**Figure 1B**). The middle segments of Arabidopsis and rice LIP5 proteins are less conserved but both contain multiple Thr or Ser residues immediately preceding a Pro residues (TP or SP, **Figure 1B**), which are the phosphorylation sites of stress-responsive MAPK3/6 important for stress-induced LIP5 protein stability in Arabidopsis (Wang et al., 2014; Wang et al., 2015).

**Figure 2:**
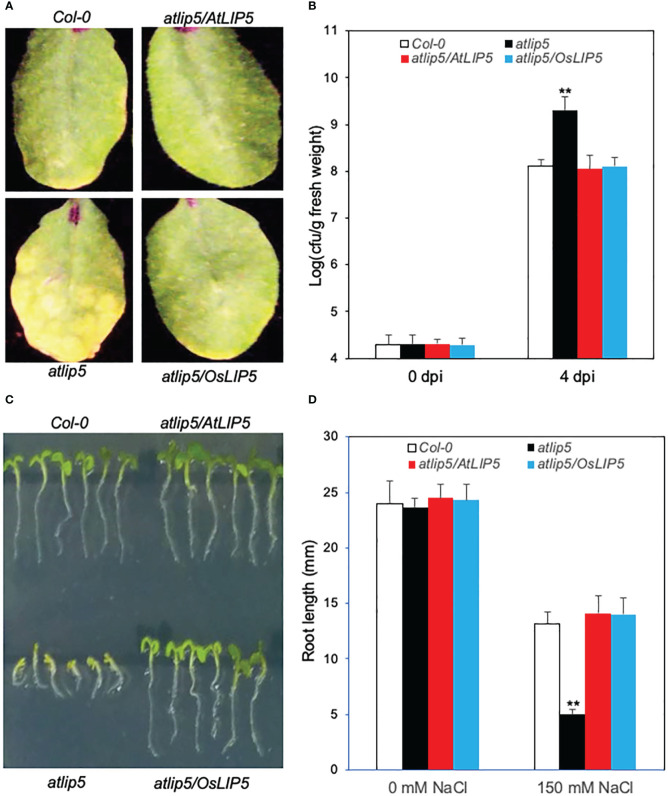
Complementation of Arabidopsis *atlip5-1* mutant by rice *OsLIP5*. Disease symptom development after infection by the virulent *P. syringae* pv *tomato* DC3000 (*Pst*DC3000). Arabidopsis Col-0 WT, *atlip5-1* and *atlip5-1/AtLIP5* and *atlip5-1/OsLIP5* lines with similar levels of LIP5-myc proteins were inoculated with *Pst*DC3000 (OD_600_ = 0.0002 in 10 mM MgCl_2_). Pictures of representative leaves were taken at 4 days post inoculation (dpi) **(A)**. Leaf samples were taken at 0 or 4 dpi to determine the bacterial growth **(B)**. The means and standard errors were calculated from 10 plants for each mutant. Double asterisks indicate statistically significant difference at P<0.01 in the colony forming units (cfu) per gram leaf fresh weight between Col-0 WT and atlip5 mutant lines determined at the same dpi. Arabidopsis seeds for the above genotypes were surface sterilized and sown on one-half-strength MS medium supplemented with 150mM NaCl. Photographs were taken 2 days post stratification and 5 days post germination **(C)**. Root length of seedlings determined at 5 days post germination **(D)**. Means and standard errors were calculated from three experiments. Double asterisks indicate statistically significant difference at P<0.01 in root length between Col-0 WT and *atlip5* mutant lines with the same salt treatment.

To compare OsLIP5 and AtLIP5 for interaction with AtSKD1, we co-transformed yeast cells with a combination of pBD-AtSKD1 bait construct with pAD-AtLIP5, pAD-OsLIP5 or pAD empty prey vector. As shown in **Figure 2C**, yeast cells co-transformed with pBD-AtSKD1 bait and pAD empty prey vectors had very low levels of *LacZ* reporter gene expression based on the β-galactosidase activity. On the other hand, yeast cells co-transformed with pBD-AtSKD1 bait and pAD-AtLIP5 or pAD-OsLIP5 prey vectors contained similarly high β-galactosidase activity (**Figure 1C**). These results indicate that like AtLIP5, OsLIP5 interacts strongly with AtLIP5.

To determine the subcellular localization of OsLIP5, we transiently expressed the OsLIP5-GFP construct in *N. benthamiana* and observed the GFP signals with confocal florescence microscopy. As previously observed with AtLIP5-GFP, the florescent signals from the leaves expressing *OsLIP5-GFP* were largely cytosolic and diffusive. However, when co-expressed with AtSKD1, OsLIP5-GFP produced a large number of punctate florescent signals and approximately 60% of these signals were also labeled with co-expressed ARA6-mRFP MVB maker signals (**Figure 1D**). These results indicate that like AtLIP5, OsLIP5 is associated with MVBs in the presence of SKD1.

### Complementation of Arabidopsis *atlip5* mutant

To determine whether rice OsLIP5 is a functional homolog of AtLIP5, we analyzed whether rice *OsLIP5* could rescue the defects of Arabidopsis *atlip5-1* mutant in disease resistance and salt tolerance. We generated the rice *OsLIP5* coding sequence fusion with a myc tag (*OsLIP5-myc*) and expressed it in Arabidopsis *atlip5-1* mutant under control of the *CaMV 35S* promoter. After infection with a virulent strain of the bacterial pathogen *Pseudomonas syringae*, the *atlip5* mutant plants developed stronger disease symptoms and supported higher bacterial growth than wild-type (WT) plants (**Figures 2A, B**). Expression of *OsLIP5-myc* completely restored the levels of resistance of the *atlip5* mutant to the WT levels (**Figures 2A, B**). When grown at 150 mM NaCl, both the cotyledon expansion and root growth of the *atlip5* mutant were greatly reduced when compared to those of WT (**Figures 2C, D**). Again, this compromised salt tolerance of *atlip5* was completely rescued by *OsLIP5-myc* (**Figures 2C, D**). At similar protein levels, OsLIP5 was as effective as AtLIP5 in restoring the disease resistance and salt tolerance of the *atlip5* mutant (**Figure 2**; **Supplemental Figure 1**). These results indicate that rice OsLIP5 is a functional homolog of Arabidopsis AtLIP5.

### Generation and characterization of rice *oslip5* mutants

**Figure 3:**
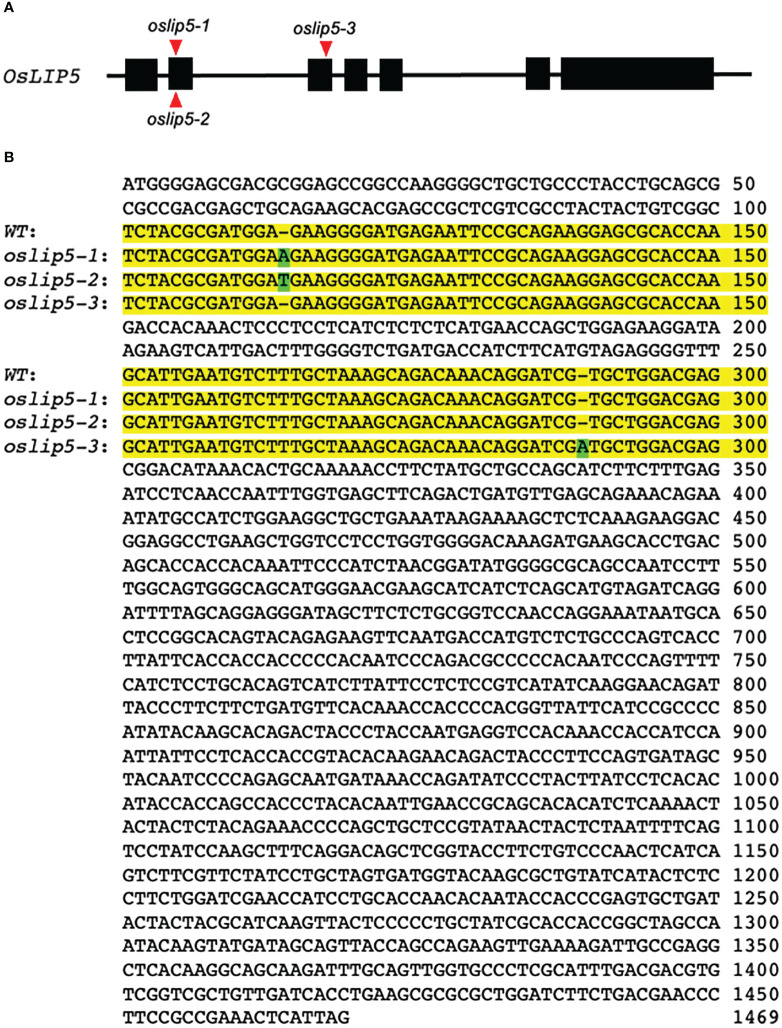
Targeted mutations of rice *OsLIP5* gene using CRISPR/cas9 genome editing. **(A)** Rice *OsLIP5* gene structure and the positions of three *oslip5* mutations on their genomic sequences as indicated by red arrowheads. **(B)** Comparison of OsLIP5 coding sequences in WT and three *oslip5* mutants. The two target sides containing the mutations are in yellow background. Inserted basis in the three *oslip5* mutants are in green background.

**Figure 4:**
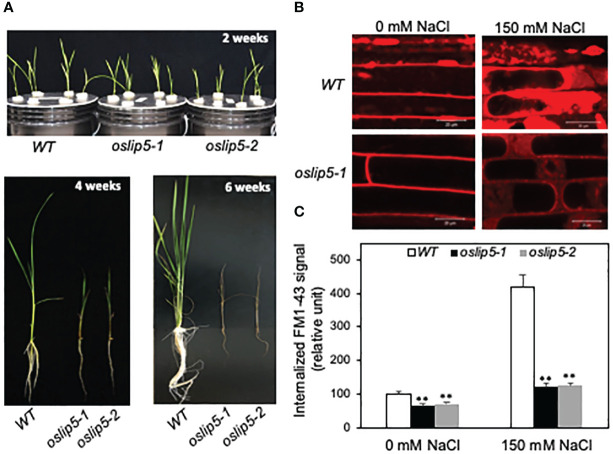
Phenotypes of rice WT, *oslip5* mutants in growth and endocytosis. Comparison of growth of 2-, 4- and 6-week-old rice WT, os*lip5-1* and os*lip5-2* mutants grown hydroponically under normal growth conditions **(A)**. Five-day-old rice seedlings were treated with 0 or 150 mM NaCl for 20 h. Basal and salt-induced endocytosis was analyzed using FM1-43 as indicated by the representative confocal images of rice root epidermal cells after FM1-43 staining **(B)**. Bars =20 μm. The fluorescent signal intensity of internalized FM1-43 was determined from 20 Z stack images of root epidermal cells **(C)**. Means and SE were calculated from images of 10 independent roots (20 images per root). Double asterisks indicate statistically significant difference at P<0.01 in the levels of internalized FM1-43 signal between Col-0 WT and *atlip5* mutant lines with the same salt treatment.

To analyze the function of rice OsLIP5 directly, we generated rice *oslip5* mutants by targeting two sites at the N-terminal domain of the OsLIP5 protein using CRIPR/cas9-mediated genome editing. We obtained three heterozygous mutants (*oslip5-1^+/-^, 2^+/-^ and 3^+/-^*) that each contain a single base insertion at one of the two target sites (**Figure 3A**). The *oslip5-1* and *2* mutants contain an A and T insertions, respectively, between nucleotides 113 and 114 of the *OsLIP15* coding sequence (**Figure 3B**), which causes a reading frame shift and introduces a premature termination codon that is expected to produce a protein of 66 amino acid residues. The *oslip5-3* contains an A insertion between nucleotides 288-289 of the *OsLIP15* coding sequence, which causes a reading frame shift and introduces a premature termination codon that is expected to produce a protein of 115 amino acid residues (**Figure 3B**). The growth and development of all these three T1 heterozygous *oslip5^+/-^* mutants were normal. In the T2 generation, three expected genotypes (WT, *oslip5^+/-^* and *oslip5^-/^* ^-^) were segregated close to the expected 1:3:1 ratio for each mutant. The homozygous *oslip5^-/^* ^-^ mutants germinated normally and grew similarly as WT during the first week post germination. However, starting from the second week post germination, the *oslip5^-/^* ^-^ mutant seedlings became reduced in size relative to WT (**Figure 4A**). With increased age, the stunted growth of the *oslip5^-/^* ^-^ mutants became more pronounced based on the increased difference of their sizes from those of WT (**Figure 4A**; **Supplemental Figure 2**). After 3 weeks post germination the leaves of the *oslip5^-/^* ^-^ mutant seedlings also developed necrotic lesions, which spread rapidly during the following 2-3 weeks (**Figure 4A**; **Supplemental Figure 2**). All the *oslip5^-/^* ^-^ mutant seedlings died in 6-8 weeks post germinations (**Figure 4A**) and, therefore, the mutations could only be maintained in the *oslip5^+/^* ^-^ heterozygous state. These *oslip5^-/^* ^-^ mutant seedlings were grown hydroponically or in soil in greenhouses or growth chambers under highly favorable conditions but they were always stunted in growth, developed lesions and all died before flowering. The strong growth defects and premature death of the *oslip5^-/^* ^-^ mutants were observed in the subsequent generations. Thus, unlike Arabidopsis AtLIP5, rice OsLIP5 is required for rice growth and survival even under normal growth conditions.

### Role of rice OsLIP5 in basal and stress-induced endocytosis

As late endosomes in the endocytic pathway, MVB biogenesis directly affects endocytosis (Stahl and Barbieri, 2002). Using the styryl dye FM1-43 as a fluorescent endocytosis marker, we have previously demonstrated that the basal endocytosis under normal growth conditions was largely normal in the Arabidopsis *atlip5* mutants (Wang et al., 2014; Wang et al., 2015). However, in salt-treated roots, the endocytic activity was induced by about 3-fold in Arabidopsis WT but only about 30% in the *atlip5* mutants (Wang et al., 2015). Likewise, pathogen-induced endocytosis was greatly compromised in the Arabidopsis *atlip5* mutants (Wang et al., 2014). These results indicate that Arabidopsis AtLIP5 is a key regulator for pathogen- and stress-induced MVB biogenesis upon exposure to pathogens and abiotic stress. The role of Arabidopsis AtLIP5 as a specific activator of stress-induced MV biogenesis and endocytosis is consistent with the normal growth and development but compromised disease resistance and stress tolerance of the *atlip5* mutants (Wang et al., 2014; Wang et al., 2015). Because of the drastic difference in the growth phenotypes of the *lip5* mutants between Arabidopsis and rice, we also compared rice WT and *oslip5* mutants for both basal and stress-induced endocytosis using FM1-43 as a marker. The membrane-selective FM1-43, which fluoresces only in a lipid-rich membrane, can enter the cells by endocytic vesicles derived from the plasma membrane (Schikorski, 2010; Amaral et al., 2011). Initial attempts to analyze the endocytic activity using FM1-43 in rice sheaths generated unreproducible results likely due to wounding and other stresses generated during FM1-43 infiltration and preparation of sheath slices. We subsequently compared WT and *oslip5* mutant roots for internalized FM1-43 signal after 20 minutes of incubation with or without prior salt treatment. Importantly, we observed significantly higher levels of internalized FM1-43 signal in WT roots than in the *oslip5* mutant roots even without salt treatment (**Figures 4B, C**). With salt treatment (at 150 mM NaCl), the intensity of internalized FM1-43 fluorescence signal increased by almost 4-fold in WT roots but only by about 35% in the *oslip5* mutant roots (**Figures 4B, C**). Thus, unlike in Arabidopsis, where mutations of *AtLIP5* affected only stress-induced endocytosis (Wang et al., 2014; Wang et al., 2015), the mutations of *OsLIP5* impacted both basal and stress-induced endocytosis in rice. Thus, the differential growth phenotypes of the *lip5* mutants are correlated with the differential roles of LIP5 proteins in the basal endocytosis under the normal growth conditions between Arabidopsis and rice.

What could be the molecular and evolutionary basis for the differential roles of LIP5 in plant growth and endocytosis in different plants? In Arabidopsis *atlip5* mutant roots, despite normal basal endocytosis when assayed using FM1-43 as marker, there are defects in the trafficking of auxin efflux transporters (Buono et al., 2016), indicating that even Arabidopsis AtLIP5 has a role in the trafficking and turnover of specific plasma membrane proteins under normal growth conditions. Besides LIP5, there are other regulators of SKD1 and MVB biogenesis such as IST1-like proteins in Arabidopsis and the lack of strong growth phenotype of Arabidopsis *atlip5* mutants could be due to functional redundancy among these MVB regulators. Consistent with this possibility, while Arabidopsis *atlip5* and *atist1-like1*(*atistl1*) single mutants are largely normal in growth and development, the *atlip5*/*atistl1* double mutant is severely stunted in growth, develops spontaneous cell death and cannot produce seeds (Buono et al., 2016). Arabidopsis LIP5 also functionally interacts with FYVE4, a plant-unique ESCRT component and regulator of MVB biogenesis (Liu et al., 2021). An Arabidopsis *fyve4* null mutant displays modestly reduced growth and early senescence but its double mutant with *atlip5* is seedling lethal (Liu et al., 2021). It remains to be determined whether the patterns of functional interactions among different regulators of MVB biogenesis might vary in different plants. If OsLIP5 acts as a major regulator of MVB biogenesis under normal growth conditions with relatively low functional redundancy with other MVB regulators in rice, its loss-of-function would have an important effect on rice growth and reproduction as observed in the *oslip5* mutants.

The drastically different phenotypes between the Arabidopsis and rice *lip5* mutants could also be attributed to the diversity among different plant species in their adaptability to perturbed cellular homeostasis. it is known that MVB pathway and related autophagy participate in plant stress responses by targeting vacuolar degradation of damaged, harmful and unwanted proteins and cellular constituents, which are often elevated under biotic and abiotic stress conditions (Li et al., 2018; Luo et al., 2021). Under normal growth conditions, however, cells also generate damaged, harmful and unwanted cellular constituents, which require prompt turnover for robust plant growth and development. In the endoplasmic reticulum (ER), about 30% of all proteins produced are misfolded under homeostatic conditions (Fink, 1999), which are mostly degraded by ER-associated degradation in the proteasome. In chloroplasts, the process of photosynthesis leads to production of damaged proteins under normal growth conditions, which are removed by both intraplastidic proteases and extraplastidic pathways including autophagy (Izumi and Nakamura, 2018). In different plant species, there could be divergence in the complex cellular networks that function in maintaining cellular homeostasis to promote growth and development. In addition, while Arabidopsis is a wild plant, rice is a crop plant that has been intensively selected artificially to increase yield and other human needs and may have evolved heighten levels of the vacuolar degradation pathways to promote growth and reproduction.

Autophagy is also a conserved degradation pathway closely related to MVB-dependent endocytosis by delivering cytoplasmic components to the lysosome/vacuole for degradation (Cui et al., 2018). In Arabidopsis, like LIP5-activated MVB pathway, autophagy is stress-inducible and autophagy mutants are strongly compromised in disease resistance and stress tolerance. Despite extensive alterations in transcriptomes, proteasomes and metabolomes, Arabidopsis autophagy-deficient mutants are largely normal in growth and reproduction (Masclaux-Daubresse et al., 2014; Mcloughlin et al., 2018; Have et al., 2019; Mcloughlin et al., 2020). In rice, however, autophagy mutants are male sterile due to defects in degradation of the tapetum and pollen maturation (Kurusu et al., 2014). Interestingly, while Arabidopsis AtLIP5-mediated MVB pathway plays a critical positive role in disease resistance, rice LRD6-6 regulates MVB-mediated trafficking to inhibit biosynthesis of antimicrobial compounds and immunity in rice (Zhu et al., 2016). Therefore, the two most extensively analyzed model plants appear to differ greatly in the way by which these two conserved vacuolar degradation pathways participate in plant growth, development and stress responses. Further analysis of the strong plant diversity in coping with perturbed cellular homeostasis could provide important new insights into the genetic and molecular basis important for both plant survival under stress condition and plant growth and productivity under normal growth conditions.

## Data availability statement

The original contributions presented in the study are included in the article/**Supplementary Material**. Further inquiries can be directed to the corresponding author.

## Author contributions

ZC and CZ conceived the project and designed the research. MW and SL performed most of the experiments. BF performed some of the experiments. MW, SL and ZC wrote the manuscript. All authors contributed to the article and approved the submitted version.
