# Supplementary material for: LIP5, a MVB biogenesis regulator, is required for rice growth
Source: Front Plant Sci. 2023 Jan 17;14:1103028. doi: 10.3389/fpls.2023.1103028 (PMC9887185; doi:10.3389/fpls.2023.1103028)
Supplement: Supplementary file 1 [file DataSheet_1.pdf]

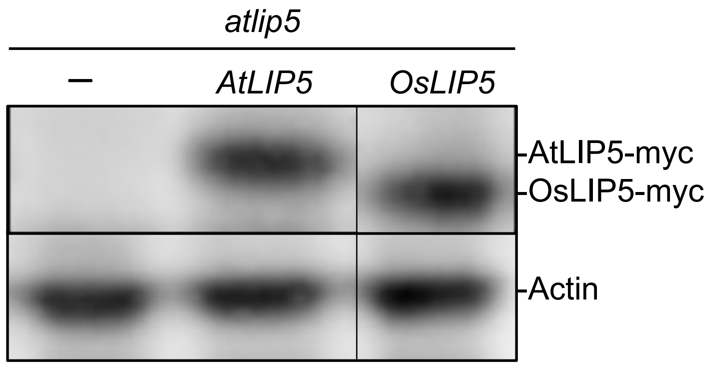

Supplemental Figure 1. Protein blot analysis of transgenic Arabidopsis *atlip5-1* plants expressing Arabidopsis AtLIP5-myc or rice OsLIP5-myc.

Proteins were isolated from the transgenic Arabidopsis plants and analyzed by protein blotting with an anti-myc antibody. Proteins from the *atlip5-1* plants were used as a negative control. Arabidopsis Actin proteins detected with an anti-actin antibody were also shown as loading controls.

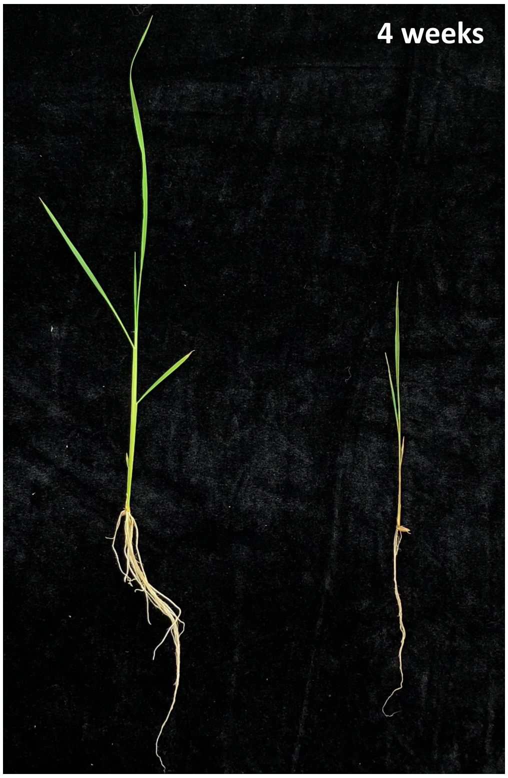

WT *oslip5-3*

Supplemental Figure 2. Comparison of growth of 4-week-old rice WT and os*lip5-3* mutants grown hydroponically under normal growth conditions.
